# Implementing a challenge-based learning experience in a bioinstrumentation blended course

**DOI:** 10.1186/s12909-024-05462-7

**Published:** 2024-05-08

**Authors:** Alejandro Santos-Díaz, Luis Montesinos, María Barrera-Esparza, Maria del Mar Perez-Desentis, David E. Salinas-Navarro

**Affiliations:** 1 School of Engineering and Sciences, Tecnologico de Monterrey, Mexico City, Mexico; 2 Institute of Advanced Materials for Sustainable Manufacturing, Tecnologico de Monterrey, Mexico City, Mexico; 3 Hospital Angeles Lomas, Mexico City, Mexico; 4 Electrónica y Medicina S.A. (EYMSA), Mexico City, Mexico; 5 Aston Business School, Aston University, Birmingham, UK

**Keywords:** Biomedical engineering, Bioinstrumentation, Challenge-based learning, Higher education, Educational innovation

## Abstract

**Background:**

Bioinstrumentation is essential to biomedical engineering (BME) undergraduate education and professional practice. Several strategies have been suggested to provide BME students with hands-on experiences throughout the curriculum, promoting their preparedness to pursue careers in industry and academia while increasing their learning and engagement. This paper describes the implementation of challenge-based learning (CBL) in an undergraduate bioinstrumentation blended course over the COVID-19 pandemic.

**Methods:**

The CBL experience was implemented in a third-year bioinstrumentation course from the BME program at Tecnologico de Monterrey. Thirty-nine students enrolled in two sections formed fourteen teams that tackled blended learning activities, including online communication, lab experiments, and in-person CBL activities. Regarding the latter, students were challenged to design, prototype, and test a respiratory or cardiac gating device for radiotherapy. An institutional student opinion survey was used to assess the success of our CBL implementation.

**Results:**

Student responses to the end-of-term survey showed that they strongly agreed that this course challenged them to learn new concepts and develop new skills. Furthermore, they rated the student-lecturer interaction very positively despite the blended format. Overall, students assessed their learning experience positively. However, implementing this CBL experience required a substantial time increase in planning, student tutoring, and constant communication between lecturers and the industry partner.

**Conclusion:**

This work provides an effective instance of CBL for BME education to improve students’ learning experience despite decreased resource efficiency. Our claim is supported by the student’s performance and the positive feedback from our industrial partner.

**Supplementary Information:**

The online version contains supplementary material available at 10.1186/s12909-024-05462-7.

## Background

Bioinstrumentation, also known as biomedical instrumentation, is considered an essential component of biomedical engineering (BME) undergraduate education and professional practice [1–3]. Bioinstrumentation entails the development of technologies for diagnostic and therapeutic purposes, such as heart rate monitors, defibrillators, and pacemakers [3, 4]. Its activities include developing sensors to capture a biosignal of interest and designing electronic circuits for amplification and filtering to build complete instrumentation systems, such as vital signs monitors [4].

Several mechanisms have been identified to provide BME students with hands-on experiences throughout the curriculum, promoting their preparedness to pursue careers in industry or academia while increasing their learning and engagement [5]. These mechanisms include computer simulation, laboratory experiments, design courses, guest speakers, industry-sponsored design projects, field trips to hospitals and medical device companies, and internships [5]. For instance, computer simulations enable students to model biomedical systems and compare theoretical performance to experimental observations. Laboratory experiments allow step-by-step exploration of physics and biological measurement principles while fostering investigation of open-ended clinical or research problems. Guest speakers provide specialized expertise and highlight the importance of interdisciplinary teamwork in biomedical problem-solving. Industry-sponsored projects, field trips, and internships offer students practical, real-world experience in clinical settings. These active learning approaches enhance student performance across science, engineering, mathematics, and social science disciplines [6, 7].

Furthermore, some of the instructional methods falling under the umbrella of Inquiry-Based Learning (IBL) have been proposed to improve BME education, including Project-Based Learning (PBL) and Challenge-Based Learning (CBL) [8–13]. IBL methods engage students in collaboratively developing solutions to real-world problems revolving around crucial concepts in the discipline, thus fostering disciplinary knowledge and creative thinking skills. IBL takes on various forms depending on factors such as the nature of inquiry, level of guidance, learning priorities, and scale [14]. It is structured inquiry when teachers provide an issue and outline for addressing it; guided inquiry, when teachers provide questions for exploration but students self-direct their inquiry; and open inquiry, when students formulate questions and control the entire inquiry cycle. In CBL, students typically engage in structured inquiry, beginning with presenting a question or real-world challenge. This is followed by the establishment of a problem-solving framework and the formulation of a plan for solution development and implementation [15].

Moreover, these methods have been found to increase student motivation and awareness of the connections between their in-class experiences and their future work [10, 16]. Referring to CBL, a distinctive feature of this approach is that the problem presented to the students has global importance or relevance (i.e., the challenge) as it provides a platform for a situated learning experience doing real things, which is argued to increase student engagement [16]. Moreover, these methods increase learning effectiveness and duration because they emphasize purposeful learning-by-doing activities in contrast with passive approaches focusing on a broadcasting type of education where students sit and listen [17].

The literature in BME highlights examples of learning challenges and CBL, demonstrating the link between real-world problem-solving and healthcare practice to enhance student learning. For instance, Martin et al. [10] address reducing severe scald burns in restaurants caused by spilled hot beverages, while Giorgio and Brophy [8] discuss designing a bioreactor for maximizing cell growth. Jansen et al. [18] also focus on tissue optics in laser treatment for port wine stains. These studies show improved student understanding, participation, and attention, leading to more fruitful learning experiences. However, our literature review shows limited studies on CBL implementations for biomedical engineering education.

This paper details the process and outcomes of implementing a CBL experience within an undergraduate bioinstrumentation blended course. The primary aim is to disseminate insights and practical knowledge from this educational endeavor, providing a reference for educators and practitioners in the field. By sharing our approach and the resultant student feedback, the paper contributes to the broader discourse on effective teaching strategies in biomedical engineering education, particularly in adapting to blended learning environments. Section 2 further details the course context, structure, and format. It also describes the role of an industry training partner in the process and the challenges presented to the students. Section 3 presents the outcomes of this CBL-based course and the results from an end-of-term survey assessing the student learning experience. Finally, Section 4 discusses these results.

### Instructional approach

CBL is a pedagogical approach where students and educators collaborate to generate questions, explore topics, devise solutions, and address compelling issues in real-world contexts [15]. This method emphasizes reflection on learning outcomes, actions’ consequences, and solutions’ communication to broader audiences. CBL is rooted in multidisciplinary work, innovation, research, and entrepreneurship. It engages students in relevant real-world situations involving defining challenges, problem-solving, decision-making, and implementing solutions.

Learning challenges represent obstacles or barriers in community, organizational, societal, or global contexts. They provide opportunities to achieve learning objectives, produce learning outcomes, develop competencies, and build learning relevance [19]. This viewpoint offers a chance to transition from conventional education toward embracing value-creation methodologies that foster the development of personal, academic, and professional skills in students [20].

CBL education has important curricular and evaluative implications as the focus is on demonstrating the pertinence and relevance of learning and academic competence, moving away from credit-hour-based programs [21]. Therefore, students benefit from increased contextual awareness, creativity, participation, reflection, applicability of learning, communication, and social interaction. Rooted in active learning, CBL involves a phenomenon perception, data collection, analysis, conceptualization, conclusion elaboration, and experimentation [16]. Accordingly, CBL develops learning achievements, evidenced by academic products and personal growth. Formative and summative evaluations occur throughout the challenge execution, utilizing various assessment tools such as rubrics, diaries, portfolios, tests, presentations, and reports.

The CBL framework provides a structured progression for identifying concerns, defining challenges, conducting problem-solving, and presenting solutions. This framework is commonly presented in terms of (i) a big idea as a broad concept that can be explored in multiple ways, (ii) an essential question to identify what is important to know, (iii) the challenge to create a specific answer or solution that can result in concrete, meaningful action, (iv) solution development concerning a thoughtful, concrete, actionable, clearly articulated, and presented alternative, (v) the solution assessment regarding connection to the challenge, accuracy of the content, clarity of communication, applicability for implementation, and efficacy of the idea, among others, and (vi) publishing results to document the experience and share with a larger audience [15].

Nevertheless, one critique of Challenge-based Learning (CBL) is its potential lack of structure and guidance, leading to ambiguity and frustration for students. The approach requires significant time and resources for planning and execution, posing challenges for educators in resource-constrained environments [22]. Additionally, while CBL aims to promote interdisciplinary learning and innovation, it may overlook the depth of subject-specific knowledge [23]. Success also relies heavily on effective facilitation from educators, without which students may struggle to engage meaningfully with the learning process. While CBL offers valuable real-world applications and collaborative learning opportunities, addressing these challenges is crucial to maximizing its effectiveness.

## Methods

The learning experience was implemented at Tecnologico de Monterrey (hereafter referred to as Tec), a private non-profit university in Mexico that recently launched its novel educational model named Tec21 Model. It provides competency-based education (CBE) grounded on the design of learning experiences to promote the development of disciplinary and transversal skills that will allow students to face the challenges and opportunities of the 21st century [24].

The Tec21 Model relies on three main pillars. Firstly, flexible and personalized programs of study. Each program curriculum is divided into three stages: (i) Exploration, a three-term stage (1.5 years) in which students get the foundations and skills of a discipline (e.g., engineering). At the end of this stage, students are expected to choose a specific academic program (e.g., biomedical engineering); (ii) Focus, a second three-term (1.5 years) stage where students are presented with core knowledge of the chosen academic program; and (iii) Specialisation, a two-term stage (one year) in which students dive into their area of specialization (e.g., biomedical image and signal analysis). Various learning opportunities are available for the students to achieve this specialization. Among them are minor and internship programs, undergraduate research, and study abroad experiences.

Secondly, CBL is the core pedagogical approach of the Tec21 Model. Its main principle involves students working with stakeholders to define an authentic, relevant challenge related to their environment, in which they will collaborate to develop a suitable solution. In addition, other active learning strategies, such as research-based learning and problem-based learning, are also used in some courses.

Finally, the third pillar of the Tec21 Model relies on inspirational professors, defined as professors who are experts in their field and actively engaged in research and professional activities. They are responsible for identifying the challenges to be tackled by students and creating the appropriate learning environments that will trigger the development of disciplinary and transversal skills.

In particular, the reported learning experience, integrated through a CBL approach, was designed and implemented in a bioinstrumentation course that is a pivotal component of our institution’s Bachelor of Science in Biomedical Engineering program. This course, positioned in the fifth term, aligns with the “Focus” stage of the program, where the curriculum is crafted to deepen students’ core competencies in biomedical engineering.

Our CBL intervention is vital in transitioning students from foundational knowledge to more complex, application-oriented learning during this stage. By confronting and addressing an industry-relevant problem, students are encouraged to apply their accumulated knowledge and skills.

The strengths of our CBL intervention are twofold. It primarily promotes active engagement with the course material, encouraging students to move beyond passive absorption of information to active problem-solving and critical thinking. This active engagement is crucial in the “Focus” stage, as it prepares students for the subsequent “Specialisation” phase and their future professional endeavors. Moreover, the CBL approach fosters a collaborative learning environment where students work in teams to navigate and solve complex problems. This collaborative aspect enhances interpersonal and communication skills and mirrors the multidisciplinary teamwork they will likely encounter in their careers. By integrating the CBL experience, we endeavor to equip our students with the necessary skills and confidence to tackle the challenges they will face in the rapidly evolving field of biomedical engineering.

Below are further details about the course context, objectives, and structure, the proposed learning experience definition and structure, the assessment tools used to collect data about its impact, and the statistical methods used to analyze the data.

### Course context, structure and format

The bioinstrumentation course is required for third-year students in the Bachelor of Science in Biomedical Engineering program. This block course runs for five weeks, 16 h per week, and includes lectures, laboratory experiments, and CBL activities. This four academic credits course is organized into four modules (Table 1). The first three modules cover fundamental concepts, circuits, and applications in bioinstrumentation and are delivered through lectures and laboratory experiments (Table 2). The fourth module entails students addressing a challenge defined by an industry partner (see subsections 2.2 and 2.3 for details). Notably, challenge-related activities are assigned 47.5% of the total course time (38/80 hours). Further details on the course’s learning contents are presented in the supplementary materials.

The CBL experience reported here was implemented in the autumn 2021 course offering. Thirty-nine students enrolled in two sections and were grouped in teams of two or three to work on assignments, laboratory experiments, and CBL-related activities. Three instructors delivered the course in team teaching, but only two were assigned to each section. In other words, one of them was assigned to both sections, whereas the other two participated in one section each.

Moreover, this course was delivered in a blended format, considering the restrictions imposed by the COVID-19 pandemic. Blended learning refers to the type of education in which students learn through different media types, using electronic, web-based, and multimedia alternatives and face-to-face traditional in-classroom options [25]. Namely, lectures and class communication with the industry partner were held online to keep minimal face-to-face interactions. In contrast, lab experiments and other hands-on activities were held in person to allow students to develop lab skills. The latter occurred in an electronics laboratory with power supplies, signal generators, and oscilloscopes. Students were also granted access to this lab outside class to complete lab experiments and hands-on activities related to the challenge. The maximum lab capacity under COVID-19 restrictions was strictly enforced by lab staff.

**Table 1:** Bioinstrumentation course modules

Module	Description	Number of hours
I	Introduction to medical instrumentation; Instrumentation amplifier; Active filters	22
II	Biopotentials and their acquisition; Sensors and transducers	12
III	Introduction to biomedical technologies: pacemakers, defibrillators, electrosurgery units; Biomedical metrology	8
IV	Challenge	38

**Table 2:** Bioinstrumentation laboratory experiments

Lab	Description	Number of hours
1	Introduction to electronics laboratory equipment and measurements	4
2	Instrumentation amplifier	4
3	Biosignal conditioning	8

### Industry training partner description and role

Previous research has demonstrated that involving an industry training partner in CBL experiences increases their complexity and uncertainty levels. Hence, student skills development is consistently higher than traditional teaching methods [26]. Accordingly, a partnership with Compañía Mexicana de Radiología (CMR) was established to implement this CBL experience. CMR is a Mexican company established in 1973 devoted to the medical imaging industry, manufacturing radiography and fluoroscopy systems, X-ray generators, digital X-ray systems, PACS, RIS, and molecular imaging systems. It also has a partner company, Electrónica y Medicina, S.A. (EYMSA), that distributes, installs, and provides maintenance to medical equipment for several medical specialties, including radiotherapy.

CMR’s role in this CBL experience included: (i) defining a bioinstrumentation design challenge relevant to their business and the wider community and presenting it to the class (week 1); (ii) giving students midterm feedback on their progress (week 3), and; (iii) assessing their proposed solutions at the end of the term (week 5). Two R&D engineers from CMR and two field engineers from EYMSA participated in this experience.

### Challenge definition and structure

Radiotherapy is a procedure for cancer treatment using ionizing radiation to destroy malignant cells. However, irradiating tumors affected by respiratory motion (e.g., lung, breast, and liver tumors) poses a risk, as radiation might unintentionally reach healthy tissues during the procedure. Respiratory-gated radiotherapy incorporates external devices to identify the phase of the breathing cycle (e.g., inspiration and expiration) and trigger radiation beams at specific times when the tumor site is predicted to be static, thus minimizing the above risk [27]. In addition, radiotherapy has also been proposed as a noninvasive technique for cardiac ablation, a procedure that scars heart tissue to block abnormal electrical signals [28]. Similarly, cardiac-gated radiotherapy ablation aims to synchronize radiation delivery with cardiac motion to increase accuracy.

In this context, students were challenged to design, prototype, and test a respiratory or cardiac gating device, which involved designing an instrumentation system to monitor the respiratory and cardiac cycle. More specifically, they were challenged to develop a bioinstrumentation system capable of sensing a signal derived from either the respiratory or cardiac cycle, implement the corresponding signal conditioning stages, analog to digital conversion, and signal processing to synchronize the physiological cycles with the radiotherapy beam. Accordingly, the challenge module was conceptually structured in three stages, each entailing some tasks:

#### Design

(a) identifying potential biosignals of interest for respiratory and cardiac gating and their characteristics (e.g., amplitude and bandwidth); (b) identifying the appropriate transducers to measure those signals and their principle of operation; (c) identifying user needs and requirements; (d) defining target specifications; (e) describing the gating device’s initial concept using sketches and low-fidelity prototypes, and; (f) describing its system-level architecture using a block diagram.

#### Prototyping

(a) designing individual stages of the device (e.g., pre-amplification, filtering, and amplification); (b) simulating each stage using relevant software (e.g., Proteus); (c) implementing the electronic circuits and testing them individually and interconnected (verification).

#### Testing

Conducting laboratory experiments to test the device’s functionality on healthy subjects.

Students were given a choice to work on respiratory or cardiac gating. Moreover, identifying specific breathing and cardiac cycle phases was not required but strongly encouraged. Therefore, the course involved the attainment of specific learning outcomes for competencies development as follows:

- SIIT0102 - Demonstrates the functioning of engineering systems and devices: Demonstrates the functioning of engineering systems and devices in real environments with typical and atypical functioning conditions throughout theoretical and empirical evidence obtained from diverse research and computational methodologies.
- SBI0201 - Measurement of medical-biological systems: Uses measurement tools in medical-biological systems for diagnostic, follow up and treatment of disease, using appropriate metrology and lab practices in the healthcare context. Performs measurements in real and controlled environments.
- SBI0402 - Integration of frontier knowledge in biomedical devices: Develops biomedical devices for the prevention, monitoring, treatment, or rehabilitation of disease, integrating frontier knowledge in engineering and medicine.

During the development of their solutions, students received continuous feedback from the course instructors. Additionally, there was an intermediate feedback session where they presented their progress to the training partner. Since the challenge involved technological development, the intellectual property of the proposals belonged to the students; however, in the case of further development of a prototype, the training partner and the university would establish a collaboration agreement. It was expected that the students would spend 38 h developing their prototypes. Furthermore, in agreement with the elements under the CBL framework presented in Sect. 1.1, the big idea (i) corresponds to the necessity of a respiratory/cardiac gating device for radiotherapy, the essential question (ii) refers to how do I measure the respiratory/cardiac cycle? Then the challenge (iii) is to design and develop a respiratory/cardiac gating device for radiotherapy/cardiac ablation. Then the solution development (iv) carried out by the students’ designs and prototypes, and the assessment (v) performed by professors and industry partners in the final presentations. Finally, the results are published (vi) in the document presented here.

### Challenge assessment

The challenge’s assessment included three formative and two summative assessments (Table 3). Formative assessments were written reports covering different tasks from the Design and Prototyping stages of the challenge. Summative assessments included a video presentation of the device with a demonstration of its functionality, a final report capturing elements from the formative assessment, and further tasks from the Prototyping and Testing stages.

Lecturers graded and provided feedback on the formative assessments. In addition, the CMR/EYMSA team provided midterm feedback on students’ progress based on reports 1 and 2 (week 3). Finally, the video presentation was graded by the lecturers and the CMR/EYMSA team, whereas the final report was graded by the lecturers only. Notably, challenge-related assessments accounted for 48% of the final course grades.

**Table 3:** Challenge assessments

Assessment	Type	Contents	Weight (%)	Week
Report 1	Formative	Tasks (a) and (b) from the Design stage	5	2
Report 2	Formative	Tasks (c) to (f) from the Design stage	5	3
Report 3	Formative	Tasks (a) and (b) from the Prototyping stage	8	4
Video	Summative	Video presentation of the device with a demonstration of functionality	15	5
Final report	Summative	Design, prototyping, and testing of the device	15	5

### Students’ assessment of their learning experience

Quantitative and qualitative data on the student learning experience were collected at the end of the course. These data were collected using the Student’s Opinion Survey, an anonymous internal survey with six closed-ended items and one open-ended question. Closed-ended items use a 10-point rating scale, with 10 representing the highest value (e.g., the highest level of agreement). The open-ended question allows students to comment on their learning experiences in the course. Here, only three closed-ended items are presented, as these are the most relevant to challenge-based and blended learning (Table 4). Furthermore, although this survey inquires about the course as a whole, the number of hours and the weight assigned to challenge-related activities makes it reasonable to attribute a strong influence of the CBL experience on student responses.

## Results

### Challenge outcomes and observations

**Fig. 1:**
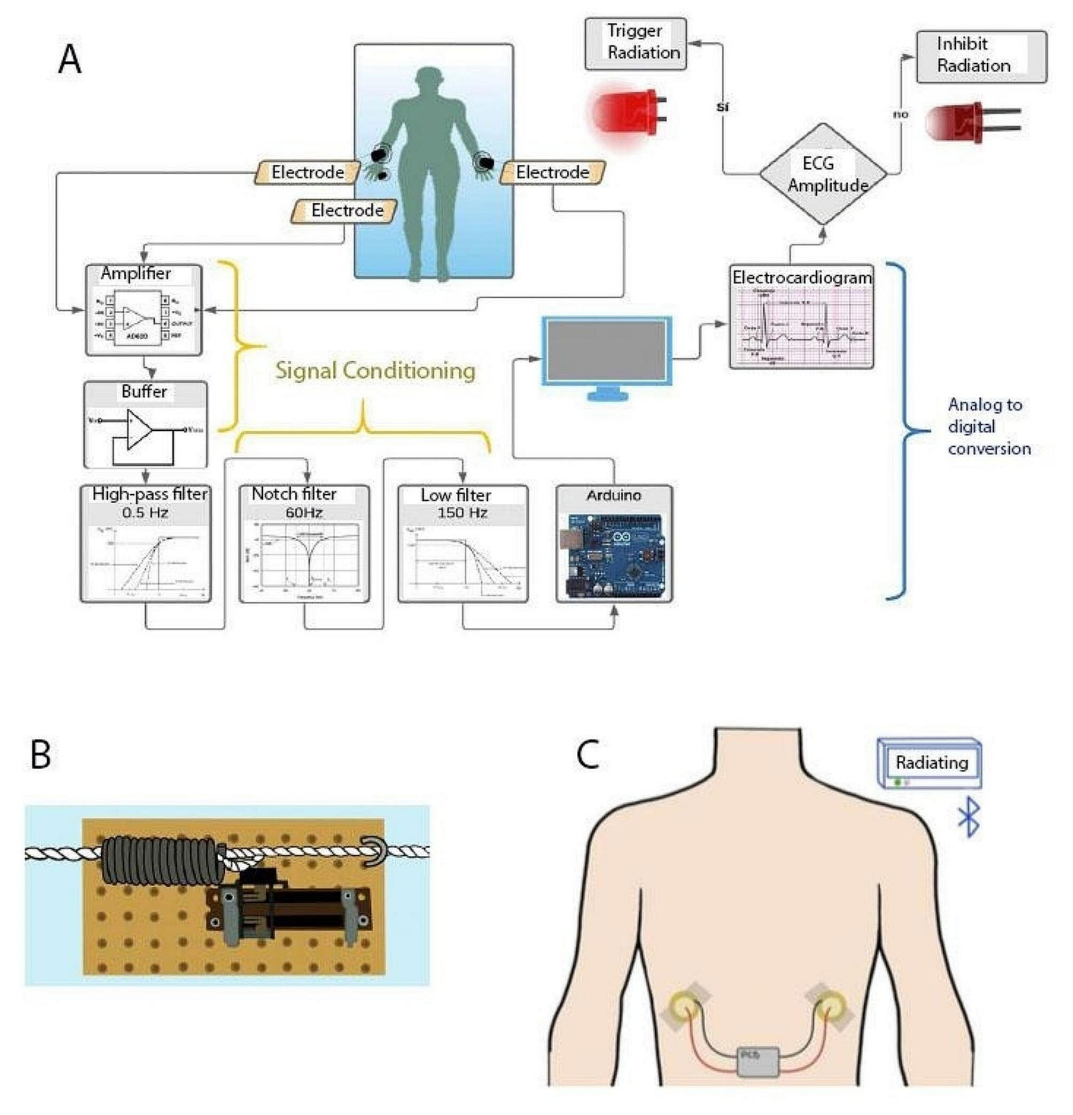
Example diagrams of some students’ designs: **(A)** ECG detection and radiation triggering using Arduino; **(B)** In-house-built mechanical transducer; and **(C)** Piezoelectric transducer array

Fourteen bioinstrumentation design projects were completed, given the class size of 39 students and their challenge work being performed in pairs or trios. Eight and six teams tackled the respiratory and cardiac gating challenges, respectively. Overall, the teams performed well in the designs they proposed. Solutions ranged from a traditional ECG measuring circuit including electrodes, pre-amplifiers, bandpass, and notch filters with A/D conversion and R wave detection using an Arduino board to respiratory gating using a temperature transducer attached to the nostril with a plastic clip. In addition, some teams presented very original ideas. For example, regarding respiratory gating, one group built their mechanical transducer using a linear variable resistor and a spring attached to a belt (Fig. 1-B). Another group used an array of piezoelectric transducers that would be attached in two lateral spots of the thorax to avoid interference with the path of the radiation beam (Fig. 1-C). Regarding cardiac gating, the solutions were similar among teams. However, the highlight was using Arduino boards to process the signal in “real-time” and detect the R wave to trigger the ablation beam (Fig. 1-A). During the final presentations, the lecturers and industry partner participants were satisfied with the students’ performance. In particular, the training partner mentioned that some ideas excelled in originality and showed “out of the box” ways of thinking. They also made students aware that, even though the prototypes were creative and interesting, the process of converting them into clinically useful tools was very extensive. However, for academic purposes, the results were satisfactory.

### Students’ assessment of their learning experience

Student responses to the Student’s Opinion Survey, with *N* = 7 for the first section and *N* = 9 for the second section, showed that they strongly agreed that this course, as delivered by their lecturers, challenged them to learn new concepts and develop new skills (EBRET), with a mean(standard deviation) score of 8.81(1.57). Moreover, they also rated the student-lecturer interaction very positively in the blended format (MHBPF), with a mean(standard deviation) score of 9.22(1.04). Overall, students’ responses showed that their learning experience was very positive (EBREC), with a mean(standard deviation) score of 9.06(1.31). Furthermore, the student comments emphasized the importance of receiving sufficient tutoring for their overall learning experience throughout the challenge.

**Table 4:** Student’s assessment of their learning experience

	Section 1 (*N* = 7)				Section 2 (*N* = 9)					
	*Lecturer 1*		*Lecturer 2*		*Lecturer 1*		*Lecturer 2*		*Pooled*	
**Item**	Mean	SD^1^	Mean	SD^1^	Mean	SD^1^	Mean	SD^1^	Mean	SD^1^
EBRET^2^	9.11	1.10	7.56	2.63	9.86	0.35	9.00	0.76	8.81	1.57
MHBPF^3^	9.11	1.10	8.67	1.41	9.57	0.73	9.71	0.45	9.22	1.04
EBREC^4^	9.33	1.05	8.56	1.89	9.57	0.73	8.86	1.12	9.06	1.31

^1^ SD: Standard deviation

^2^ EBRET: The professor challenged me to give my best (e.g., developing new skills and learning new concepts)

^3^ MHBPF: The interaction with my professor in the blended format was:

^4^ EBREC: Overall, my learning experience was:

## Discussion

The instructional approach described above led to the successful implementation of a CBL experience in a bioinstrumentation blended course. This experience incorporated elements that provide BME students with hands-on experiences relevant to their future careers, including computer simulations, laboratory experiments, and an industry-sponsored design project [5]. The participation of an industry training partner in the course was critical to successfully implementing this CBL experience. It encouraged student engagement by challenging students to tackle a problem relevant beyond the classroom. In addition, it also allowed them to learn about a field not necessarily covered in the traditional BME curriculum (i.e., radiation therapy).

Student responses to the end-of-term survey suggest that CBL actually challenges students to learn new concepts and develop new skills. They also suggest that CBL can be successfully incorporated into a bioinstrumentation blended course, given the right balance between online and face-to-face interactions. This is supported by students rating their overall learning experience very positively. In addition, students, training partners, and teachers held a rich and insightful discussion during the final evaluation session. On one hand, students commented on the challenges they faced when designing and prototyping their solutions and how they aligned well with the course contents. Also, they commented on the valuable feedback they received from our partner and how it helped them move towards improving their prototypes. On the other hand, the training partners congratulated students on their designs and prototypes. They commented on the pleasant surprise of looking at some “out of the box” solutions for the gating problem. These discussions and the overall positive ratings from students support the success of our CBL implementation in a bioinstrumentation blended learning environment.

Moving towards pedagogic techniques such as CBL in biomedical engineering enhances students’ engagement and relevance to their learning [9]. Our approach connected our students to a real-life scenario where they could relate the academic contents to a problem faced by our industrial training partner, making the bioinstrumentation course more relevant and exciting for them, as they could directly relate their learning to an aspect of their future professional practice. Similar active and experiential learning approaches have been successfully implemented in biomechanics [29], whereas bioinstrumentation has been targeted using project-based learning implementations [12]. Here, we present a case of success in implementing CBL in an undergraduate bioinstrumentation course in a blended format that combined online lecturing and in-person hands-on lab work and involved an industrial training partner. Our results, albeit primarily qualitative, support two main ideas. First, students’ learning is more significant when using a CBL approach, and second, it may be used as a seedbed for ideas with the potential to be developed in the industry.

However, implementing the CBL experience described above presented challenges for the teaching team. It required around 12 h of planning before the course and constant communication between lecturers, the teaching team, and the industry training partner. In addition, lecturers had to dedicate around 35 h to tutoring students on their design projects. These critical aspects must be considered when implementing a CBL experience in any course.

From the industry training partner perspective, it was also a challenging experience due to the necessity of providing students with the appropriate insights to implement a functional prototype in the conditions to be evaluated. The mid-term industry feedback sessions allowed students to work on key software and hardware components of their projects, thus fulfilling the prototype expectations for the application. Also, the commitment between the industry training partner and students must run both ways to achieve the highest benefit. On the one hand, students develop skills and competencies to be better prepared for their professional life. On the other hand, the industry training partner may find innovative ideas to solve a challenge they have, as students provide “fresh eyes” to the problem.

Finally, our study presents some limitations. First, since we did not compare against a control group using a more traditional educational approach, we did not run quantitative statistical analysis to proof the possible advantages of CBL in this context. Incorporating a control group—possibly another class where traditional teaching methods are employed—would allow for a more robust analysis. This setup would enable a direct comparison, highlighting the specific contributions of CBL to student performance and thereby boosting the validity of the findings. Second, we did not include responses from open-ended questions in the students’ survey since they primarily focused on the instructor’s performance rather than students’ direct experiences with CBL. This oversight limits our understanding of the subjective impact of CBL on students, including their perceptions and any specific challenges they faced. Adding some questions where students can express their opinions on the CBL implementation would enrich the qualitative assessment.

## Conclusion

This work reports the successful implementation of a CBL experience in an undergraduate biomedical instrumentation blended course that included remote lectures and in-person lab work. The collaboration with an industry training partner was key to success since students felt strongly challenged to learn and develop new concepts and skills while solving an interesting and real industry-related problem. Nonetheless, the extensive time required for course preparation and tutoring and the close communication between lecturers and industry partners are key components to be considered when implementing such experiences. Despite the blended format, we successfully implemented this CBL experience, as reported by our students’ opinions.

### Electronic supplementary material

Below is the link to the electronic supplementary material.

Supplementary Material 1

## Data Availability

All data generated or analyzed during this study are included in this published article.
