# Supplementary material for: Implementing a challenge-based learning experience in a bioinstrumentation blended course
Source: BMC Med Educ. 2024 May 8;24:510. doi: 10.1186/s12909-024-05462-7 (PMC11077816; doi:10.1186/s12909-024-05462-7)
Supplement: Supplementary file 1 — Supplementary Material 1 [file 12909_2024_5462_MOESM1_ESM.docx]

**Implementing a Challenge-Based Learning Experience in a Bioinstrumentation Blended Course**

**Alejandro Santos-Díaz ^1,*^ Luis Montesinos ^1,2,*^ , María Barrera-Esparza ^1,3^, Maria del Mar Perez-Desentis ^4^ and David E. Salinas-Navarro ^5^**

1. School of Engineering and Sciences, Tecnologico de Monterrey, Mexico City, Mexico; [alejandro.santos@tec.mx](mailto:alejandro.santos@tec.mx)
2. Institute of Advanced Materials for Sustainable Manufacturing, Tecnologico de Monterrey, Mexico City, Mexico
3. Hospital Angeles Lomas, Mexico City, Mexico; [mariabarrera@tec.mx](mailto:mariabarrera@tec.mx)
4. Electrónica y Medicina S.A. (EYMSA), Mexico City, Mexico; [mperez@eymsa.com](mailto:mperez@eymsa.com)
5. Aston Business School, Aston University, Birmingham, United Kingdom; [d.salinas-navarro@aston.ac.uk](mailto:d.salinas-navarro@aston.ac.uk)

*Correspondence: ASD; [alejandro.santos@tec.mx](mailto:alejandro.santos@tec.mx) ; LM; [lmontesinos@tec.mx](mailto:lmontesinos@tec.mx)

***Supplementary materials***

**Detailed learning contents of the Bioinstrumentation course.**

The official name of the course where the learning experience described in this article is BI2005B - Application of bioinstrumentation and biomedical technologies. This is a four academic credit block course corresponding to the fifth semester (focus stage) of the bachelor in biomedical engineering program at Tecnologico de Monterrey. The learning contents described in the curriculum are listed below:

**Module 1**

1. Introduction to medical instrumentation.
   1. Overview of medical instrumentation.
   2. General medical instrumentation system/chain.
   3. Definition of physiological variables.
2. Electronic instrumentation.
   1. Instrumentation amplifier: characteristics and application in biomedical.
   2. Isolation amplifier: characteristics and application in biomedical.
   3. Types of isolation: optocouplers, magnetic flux and capacitive couplers.
   4. Ideal sampling and reconstruction of continuous-time signals: discrete-time processing of continuous-time signals - and vice versa - maximum conversion frequency and considerations on the sampling period.
3. Operational amplifiers in linear applications.
   1. Active filter: definition, types and specifications.
   2. Filter transfer function.
   3. First and second-order functions.
   4. Butterworth and Chebyshev filters.
   5. First order physical realizations.
   6. Second-order physical realizations (KRC, feedback multiple, state variables, bi-quadratic).
   7. Oscillators.

**Module 2**

1. Biopotentials and their recording.
   1. Generation of biopotentials: cell membrane model.
   2. Electrodes and micro-electrodes (invasive and non-invasive).
   3. Instruments for capturing physiological signals: ECG, EMG, EEG and EOG.
2. Sensors and transducers.
   1. Sensors: definition and basic principles.
   2. Resistive, piezoresistive, and capacitive sensors.
   3. Inductive, optical and temperature sensors.
   4. Transducers: definitions, static and dynamic characterization.
   5. Optoisolators: definition and applications.

**Module 3**

1. Introduction to biomedical technology.
   1. Pacemakers.
   2. Defibrillators.
   3. Electrosurgery units.
   4. Electromagnetic compatibility in hospital environments.
2. Biomedical metrology.
   1. Basic concepts of metrology.
   2. Biomedical quantities.
   3. Criteria for metrological and regulatory assurance.
   4. Applications.

**Student’s opinion survey questionnaire**

In Tecnológico de Monterrey, students are compelled to answer the Student’s Opinion Survey at the end of every course. This is a standardized survey about the student’s experience in each course and with each professor, with a point scale from 0 to 10, where they represent the worst and best experience, respectively . Questions included in the survey are presented as follow:

Close ended questions included in the survey are the following:

1. EBDOM: The professor shows dominance and experience on the course topics.
2. EBRET: The professor challenged me to give my best (develop new skills, new concepts and ideas, think differently, etc.).
3. EBASE: The professor promoted an environment of trust and respect.
4. EBMET: The support I received from my professor was appropriate (answers to questions, guidance, feedback, etc.).
5. EBREC: Overall, my learning experience with the professor was:
6. MHBPF - The interaction with my professor under the HyFlex+ Tec Model (in remote, hybrid, and in-person groups) has been:

The open ended question is as follow:

1. What would you say to a student who wanted to enroll in the class with this professor?
